# Recovery in Mind: A Recovery College's journey through the Covid‐19 pandemic

**DOI:** 10.1111/hex.13635

**Published:** 2022-10-25

**Authors:** Heather Yoeli, Angela Ryan, Cath Hensby, Fiona Habermehl, Sarah Burton, Jacqueline Sin

**Affiliations:** ^1^ School of Health and Psychological Sciences City, University of London London UK; ^2^ Northern Lights Research Associates Newcastle upon Tyne UK; ^3^ Recovery in Mind Newbury UK; ^4^ Berkshire Healthcare NHS Foundation Trust Bracknell UK

**Keywords:** co‐production, Covid‐19, peer trainers, Recovery College, well‐being

## Abstract

**Introduction:**

The Covid‐19 restrictions of 2020–2021 are known to have undermined the UK population's mental health. Working alongside staff, peer trainers and students at Recovery in Mind (RiM), a Recovery College (RC) in West Berkshire, England, this mixed‐methods study is amongst the first to investigate how an RC has responded to the pandemic.

**Methods:**

Working in co‐production with RiM staff and peer‐trainers, this study employed a mixed‐methods design, gathering Warwick‐Edinburgh Mental Wellbeing Scale (WEMWBS) well‐being outcome measures by questionnaire and student experience, learning and co‐production by interviews.

**Findings:**

This research found that RiM continued to produce demonstrable improvements in student mental health. Students welcomed the way that RiM adapted to offering online and socially distanced provisions. Students valued the skills that RiM taught and the way that RiM courses reinforced prior learning; above this, however, they valued the mutual support and sense of community that participation provided.

**Conclusion:**

This study underlines the value of RCs maintaining ‘hidden curriculums’ of peer support and community involvement. This research emphasizes co‐production as not only a tool for empowerment or service improvement but as a valuable skill for personal mental health recovery. Even when operating under the most unforeseen or challenging of conditions, RCs should always endeavour to prioritize and maintain co‐production.

**Patient or Public Contribution:**

In accordance with the RC ethos, this was an entirely co‐produced study, with academic researchers and RiM staff and peer trainers working democratically in partnership with one another to design and manage the study and to write up and disseminate findings. To ensure the independence and rigour of findings, data analysis was undertaken by external academic researchers.

## 1 INTRODUCTION

The Covid‐19 pandemic and its ‘social distancing’ restrictions are known to have undermined population mental health, both globally and in the United Kingdom.^1^, ^2^, ^3^, ^4^ During 2020 and 2021, the entire United Kingdom was placed under national lockdown three times (March–June 2020, November 2020 and January–March 2021), with a complex tiered system of localized restrictions between these. The pandemic has led to increasing symptoms and declining well‐being for people with pre‐existing mental ill‐health.^5^, ^6^ Both people with and without pre‐existing mental health needs reported finding mental health support more difficult to access and engage with.^5^

To date, little research has evaluated how Recovery Colleges (RCs) or similarly user‐led or co‐produced mental health services have responded and adapted to the Covid‐19 pandemic.^7^ However, emerging evidence both globally and from the United Kingdom suggests that, from the outset of the pandemic, co‐production, broader forms of service user participation and patient and public involvement^8^ in mental health services have been significantly de‐prioritized over the last 2 years.

## 2 BACKGROUND

### 2.1 RCs

In the United Kingdom, the RC model was developed in the mid‐2000s.^9^ As of 2020, there were approximately 80 RCs across the United Kingdom, with an increasing number operating worldwide.

The RC model is guided by values of hope, control and opportunity, and aims to empower individuals and communities with the skills to improve their own personal and collective mental well‐being. RCs seek to function as educational rather than therapeutic institutions: participants are students not patients; students register for courses rather than being referred, and students attend classrooms rather than day centres.^10^, ^11^ RCs adhere to an educational philosophy of emancipation and action learning,^11^, ^12^ grounded in co‐production by valuing the lived experiences of people living with mental health challenges.^11^, ^13^

RC co‐production involves students and peer trainers.^11^, ^12^, ^14^ Peer trainers are former RC students who have been employed to co‐produce all aspects of RC planning, development, governance and delivery, drawing upon both their lived experiences of mental ill‐health and recovery,^11^, ^14^ and upon their broader personal and vocational skills.^11^, ^15^ In the same way that the model differentiates RCs from organizations providing treatment or care, so also it differentiates peer trainers from many of the peer support workers increasingly working within other forms of mental health services provision. Peer trainers are educators and mentors, not carers, and may exercise their skills at all strategic and operational levels of RC activity.^11^, ^14^ The co‐production of RCs, therefore, empowers people with lived experience not only to contribute and become involved but to assert leadership and enact change.^11^, ^13^

RCs have been extensively evaluated and researched,^10^, ^15^, ^16^, ^17^, ^18^, ^19^, ^20^, ^21^, ^22^ with systematic and narrative reviews offering a strong evidence base from which to claim that RCs are effective in improving service users' quality of life and self‐management skills, and lead to lasting organisational change within mental health services.^10^, ^23^, ^24^, ^25^ A Canadian initiative is currently co‐producing a user‐led framework for evaluating RCs.^26^ Increasingly, RC research is diversifying, exploring the experiences of students with specific diagnoses and studying the impacts of specific RC courses.^21^, ^27^, ^28^ One of the first pieces of RC research to have explored the effects of the Covid‐19 pandemic and the move online is that of Rapisarda et al.^29^ Their study found online RCs to be particularly effective at combatting anxiety, and advocates RCs as a cost‐effective and accessible public mental health response to crisis situations.^29^

### 2.2 Recovery in Mind (RiM)

RiM is an RC in West Berkshire, in rural South‐East England. It was founded in 2016 and is led by AR, an experienced voluntary sector manager with lived experience of mental ill‐health. In addition to AR, RiM's team comprises Occupational Therapy (OT) staff and peer trainers. Some of these OTs are seconded by the local NHS Trust and some are employed on a zero‐hours basis funded by Big Lottery Fund. Peer trainers are also given zero‐hours contracts which, like the salary of AR as the RiM CEO, are funded by various UK charity grants. Everyone on a zero‐hours basis is paid in excess of the local living wage and has been fully remunerated for work they would have done if not for the disruption of Covid‐19.

The study aimed to evaluate RiM's impact and its courses in promoting students' well‐being and subjective perception of recovery and to explore students' and peer trainers' lived experiences of recovery through participation in RiM. The study was integral to the RiM's expanded course delivery supported by Big Lottery Fund, and lasted over a 2‐year period, from June 2019 to June 2021. This study also served to document RiM's journey through the pandemic. This paper explores how and why RiM has adapted its courses over the pandemic and how students and peer trainers journeyed with RiM.

## 3 METHODS

### 3.1 Charting RiM's journey through Covid‐19

More than 110 students joined RiM Steps 1 and 2 courses over the study period of June 2019 to June 2021.

In March 2020, with the first lockdown in the United Kingdom enforced, RiM's initial response was to start a weekly email newsletter to communicate with all current and former students and peer trainers.

With the lockdown and restrictions on face‐to‐face contacts continuing to render it impossible to run courses for months, RiM converted the Step 1 Recovery Bitesize into a video. RiM staff produced a series of online nano‐courses and relaxation exercises as a freely accessible resource to all struggling to cope with the pandemic. Essentially, the team tried to remain flexible in their approach, and this, coupled with a ‘can do’ attitude enabled the service to respond to the pandemic. RiM came to regard the use of technology as essential in keeping communication going despite the initial reticence and anxiety from staff, peer trainers and students alike. The team had to make a number of ‘best guess’ judgments in the early days of the pandemic when online communications were still new to many. In January 2021, the team launched a regular online Coffee Catch‐Up session.

**Figure 1:**
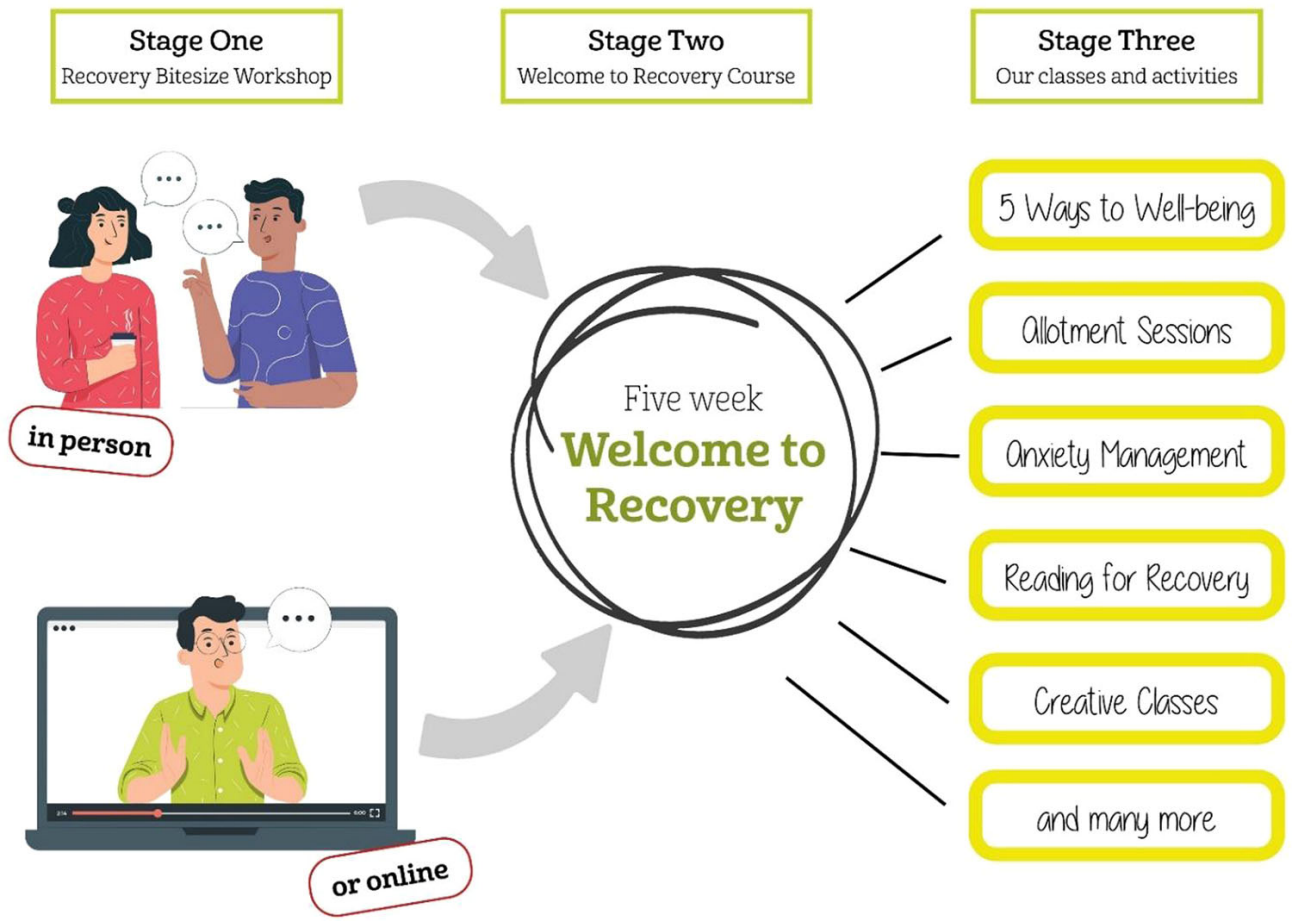
The three‐stage model of RiM

With the partial lifting of restrictions in the Summer of 2020, RiM experimented with outdoor activities built upon existing nature‐based provisions. Particularly successful were the *Walk and Talk* sessions, later renamed *Ponder and Wander*. In Autumn 2020, RiM resumed indoor face‐to‐face interaction by delivering *Welcome to Recovery* (WTR) (see Figure 1) in a larger and more Covid‐secure venue than previous premises. When the November 2020 lockdown was announced shortly after the start of a *Five Ways to Wellbeing* course at another outdoor venue, the RiM team decided to move the remainder of the course online using Microsoft Teams. In December 2020, RiM resumed some outdoor sessions, culminating in a physically distanced Festive Walk. Throughout the pandemic, all face‐to‐face activities were delivered in accordance with the Covid‐19 protocols of Berkshire Healthcare NHS Foundation Trust and in compliance with contemporaneous pandemic restrictions imposed on West Berkshire.

Following the January–April 2021 lockdown, RiM developed a number of new active and creative activities, some online, some outdoors and some held in the allotment polytunnel which RiM now shares with another community group. Approximately two‐thirds of the students started the courses face‐to‐face though more than half of them had experienced part of their WTR courses being delivered online. The remaining one‐third undertook all courses online. However, when *Recovery Bitesize* workshops were delivered as an emailed video link, no questionnaires relating to it were administered as it felt inappropriate without a face‐to‐face opportunity for the team to explain the research.

### 3.2 Co‐production of the mixed‐methods approach

**Figure 2:**
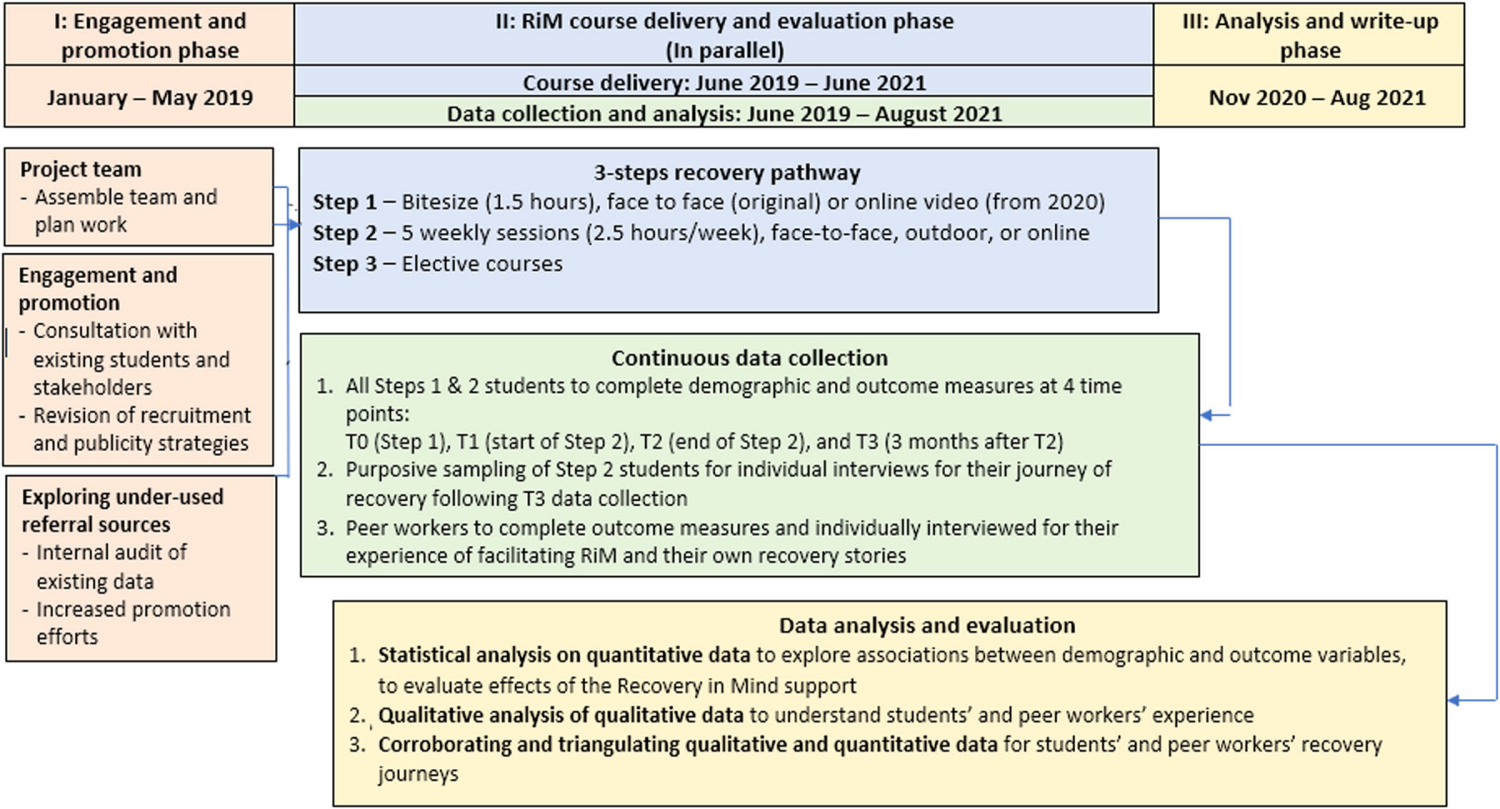
Flow chart of study design

When studying user‐led or co‐production initiatives, it is necessary for research to appropriate and extend organisational values — in the case of RCs, those of hope, control and opportunity — to amplify the voices of the historically least‐heard participants, and to challenge their marginalization.^30^ This study was co‐designed and co‐produced by researchers, RiM staff and peer trainers, who developed a mixed‐methods approach aiming to combine the advantage of quantitative and qualitative research (see Figure 2).^18^, ^31^

#### 3.2.1 The quantitative component

To explore individual students' well‐being and goal‐achievement, the research used two questionnaires:

1. The Warwick‐Edinburgh Mental Wellbeing Scale (WEMWBS)^32^ is a self‐report measure comprising 14 positively worded statements (e.g., ‘I've been feeling optimistic about the future’). Total WEMWBS scores range from 14 (minimum) to 70 (maximum); the higher the score the better the individual's positive mental well‐being. A change of three points in WEMWBS represents the minimum clinically important difference (MCID).^33^ WEMWBS has been widely used in epidemiological research since 2009, including the Health Surveys for England.^34^
2. A two‐part Personal Goal Questionnaire was devised by RiM staff and peer trainers to establish a subjective evaluation of the achievement of a personal goal. Each student specified a personal goal that they would like to achieve through the RiM courses, followed by a Likert scale of 0 (worst) to 10 (best) to rate themselves in terms of achieving that.

The researchers invited each student to complete WEMWBS and a Personal Goal Questionnaire at four time points: T0 (before their Step 1 *Recovery Bitesize* workshop); T1 (at the beginning of their Step 2 *Welcome to Recovery* (WTR) course); T2 (at the end of their WTR course) and T3 (3 months after they had completed WTR). When courses were delivered in person, questionnaires were completed by students at the beginning (T0 or T1) or the end (T2) of the respective session. T3 questionnaires were posted to students with a self‐addressed stamped envelope to return completed assessments. However, as previously explained, when *Recovery Bitesize* workshops were delivered online, no T0 questionnaires were administered. During the time of online delivery, questionnaire administration was via post and email, with telephone support provided for those who preferred to complete questionnaires over the phone.

In addition to comparing students' mental well‐being with the English population norms on the WEMWBS^34^ using independent‐sample *t*‐tests, we explored changes in students' well‐being and goal‐achievement across the time points, using descriptive statistics. Subjective ratings of personal goal achievement were analysed descriptively. Missing data on individual WEMWBS scores were dealt with by replacing up to three missing and incomplete WEMWBS items with the average value of the other item values.^35^

#### 3.2.2 The qualitative component

##### Sampling and recruitment

When the research was first designed in 2019, researchers envisaged a purposive approach within which the researchers and RiM would work together to select and recruit a sample of students who might best reflect the diversity of student demographics. However, as Covid‐19 disrupted course delivery and study recruitment, researchers moved towards a more pragmatic sampling method, inviting participation from students who had remained most engaged with RiM following its move to online provision.

##### Interviews

Semistructured topic guides for the student interviews were designed and piloted by RiM staff, peer trainers and students, working together in accordance with the co‐production ethos of RiM. Student interviews were carried out by telephone and Zoom by SB, asking participants about their RiM experiences and more generally their lived experiences of the Covid‐19 pandemic and recovery. Interviews were audio‐recorded, transcribed and subjected to thematic framework analysis^36^ using NVivo12 research software.

### 3.3 Ethics

Ethical approval for this study was obtained from the University of Reading Research Ethics Committee on 15 May 2019 (reference: UREC 19/21). Further minor amendments to adapt the data collection through online means during the pandemic were approved in March 2020. All participants signed consent forms. All participants were offered pseudonyms and outcome measures were coded anonymously.

## 4 FINDINGS

### 4.1 Participant characteristics

Eighty‐nine students completed the questionnaires at T0, at the beginning of the Bitesize course. Students' ages ranged from 18 to 78 with a mean of 47 years (standard deviation [SD] = 14, median = 47). Table 1 summarizes participant demographics.

**Table 1:** Participant demographics

	Quantitative	Qualitative
	*N* (%)	*N*
Total number of participants	89	6
Gender		
Male	26 (30)	1
Female	63 (70)	5
Ethnicity		
White British	75 (54)	5
Other White	6 (7)	1
Asian	4 (4)	0
Mixed heritage	3 (3)	0
Undisclosed	1 (1)	0
Employment status		
Employed	37 (42)	0
Self‐employed	5 (6)	0
Retired	10 (11)	3
Volunteering	3 (3)	0
Not working	33 (37)	3
Looking for work	16 (18)	0
Undisclosed	1 (1)	0
How they heard about Recovery in Mind		
GP	29 (33)	1
CMHT	27 (30)	1
Friends	10 (11)	0
Family	0	1
Publicity or social media	5 (6)	1
Other/undisclosed	17 (19)	2

### 4.2 Questionnaire findings

#### 4.2.1 Mental well‐being

At T1, 71 students completed the WEMWBS; at T2, 44; at T3, 18. Before commencing (T0), the mean mental well‐being (WEMWBS) scores across all students (*n* = 89) was 35.59 (SD 9.67). Students' WEMWBS scores ranged between 14 and 65. No significant difference in WEMWBS scores among male and female students was found (independent‐sample *t*‐test, *p* = .56). Similarly, age or employment status had no significant effects on the students' baseline WEMWBS scores, as shown by univariate analysis of variance, respectively (*p* = .77 for age, *p* = .99 for employment status).

#### 4.2.2 Comparing WEMWBS of RiM students with the general population

Students' WEMWBS scores at T0 were compared with the average WEMWBS score (49.9, SD 10.8) of the general population as reported in Health Survey England (HSE) in 2016.^34^ An independent‐samples *t*‐test showed that the mean difference of −14.31 WEMWBS scores was statistically significant between RiM students and the HSE sample (*t* = −13.85, 95% confidence interval [CI] = −16.34 to −12.29, *p* < .001). This difference is nearly five times the MCID of 3 points on WEMWBS.^33^ WEMWBS has been compared to the Centre for Epidemiological Studies Depression Scale (CES‐D), a measure of depression^37^ which suggests that scoring less than or equal to 40 on WEMWBS indicates a high risk of clinical depression. The sample average of WEMWBS of 35.59 indicated a significant likelihood of depression or distress amongst students.

#### 4.2.3 Changes in students' WEMWBS scores over time

At T2, students' WEMWBS ranged from 27 to 57 and their average WEMWBS score was 44.27 (SD 9.45). Male and female students' mental well‐being scores were similar. While these figures are still below that of the population norms, it is significantly higher than the scores before them undertaking the *Welcome to Recovery* course (difference = +8.88). The increase in WEMWBS score of 8.88 points was nearing 3 MCID, indicating a significant improvement in mental well‐being among the students through the course.

At T3, students' WEMWBS scores averaged 43.09 (SD 10.89), ranging from 25 to 57. There was little difference between the scores reported by male and female students. The gain made by students in WEMWBS scores previously appeared to be sustained in the follow‐up.

#### 4.2.4 Subjective evaluation of personal goal achievement

At the beginning of the courses, 73 students completed the personal goal achievement question. Some of the wide‐ranging personal goals the students set for themselves are detailed in Box 1.

Box 1 **Alphabetical list of student personal goals**

- Appreciate life
- Be able to talk and not bottle things up… [to avoid] the spring has been wound up so tight, and the spring breaks and explodes
- Be kinder to myself
- Feel better about myself and stop over‐thinking and also be back to my old smiley happy self again
- Gain confidence and recover my mental health
- Improve my self‐confidence
- Not rushing into decisions
- Overcome my stress
- Think clearly
- To feel I am capable in life and worthwhile and less anxious all the time
- To feel it's okay to be me and be comfortable with my emotions
- To lower my daily anxiety level
- To not be overwhelmed by stress, to be able to relax, to like myself

The baseline goal‐achievement scores ranged from 0 (not at all) to 10 (completely), with a mean score of 3.07 (SD 2.16) and a median score of 3.00. These scores indicated the students felt they had a long way to go in achieving their goals. At T2, 38 students rated their own personal goal achievement. For male students (*n* = 8), the mean score was 5.63 (SD 2.07) and the median score was 5.50. For female students (*n* = 30), the mean score was 5.03 (SD 2.08) and the median was 5.07. At T3, 16 students, 5 males and 11 females completed this question. The mean and median scores among the male students were 4.20 (SD 2.49) and 4.00 and for females were 5.18 (SD 2.71) and 6.00, respectively.

### 4.3 Interview findings

Six students were interviewed, in interviews ranging from approximately 25 to 90 min duration. The analysis identified three main themes, each with a number of subthemes.

#### 4.3.1 The skills that RiM taught

In describing the contents of their personal toolkits, students distinguished between the skills that they had been taught within RiM classes and the wellbeing strategies which RiM had empowered them to discover and develop for themselves.

##### The skills I learned from RiM and the strategies I developed for myself

Students described how they were using and applying the skills developed at RiM.

I always think when I go out for a walk … after I've done an online RiM coffee morning, whatever we've discussed in that session I find I think about and gives me good thinking time. It makes me more aware. One time we were talking about signs of spring … someone mentioned about things in the hedgerow, so I was looking there. It gives you more of a mindfulness time. (Carrie)

Students described becoming increasingly confident in pursuing these skills independently, taking increasing amounts of initiative to develop their own personal strategies for well‐being:

The Wellness Recovery Action Plan [course delivered in 2019], that inspired me in taking up tennis again, which was something that I used to do, because you learned to take up new things as well as trying to take up old things that you used to do to help your mental health recovery. (Nancy)

The isolation of the Covid‐19 lockdowns gave students a particular need to practise skills of mindfulness and community involvement more independently. Several students described how they had used the skills, strategies and social support provided to feel sufficiently confident to pursue those social opportunities less restricted by the pandemic:

I realised that I do some running but I'm always running on my own so I picked up on the idea of exercise with other people is something that I really ought to be involved in more. I've actually looked into the Sport in Mind and next week I'm going into Newbury to kick a football for a little while and I think that will be fun. (Joe)

##### How RiM reinforced what I'd learned from other mental health services

For some students, these skills and strategies were things that they had learned about in the past from other mental health treatments they had received. These students were grateful to RiM for the reminder, the reinforcement and the practical support which RiM provided for them to practise and to develop these skills and strategies:

We did personal boundaries/personal responsibilities and that I could link to my sessions with my therapist because it was like my values and the choices that I take and value, so it was quite interesting doing that again because a lot of people have similar boundaries or similar choices/values. (Heidi)

For other students, RiM was completely different from anything they had ever tried before and helped them as nothing else had.

##### Engaging with others online

Most students found online classes comfortable and effective. They observed that RiM had worked hard to facilitate and maintain what felt like a safe space and that the staff and peer trainers facilitated well together:

The team were very professional, and I found Zoom meetings to be an advantage … I mean I'm sitting here with the screen, you can just about see my head and you miss a lot of body language and stuff like that obviously (Joe)

Some, who had been less accustomed to online interactions and had been more hesitant, rapidly developed their skills and confidence:

It could have been two ways. Either I could have said, do you know what I can't do it today and not go at all or I said okay, I've got to go, I've made a commitment, it's important to go … With the online process you can actually switch off, you're still there but you can switch off completely. (Julia)

Overall, students agreed that, whereas online sessions probably cannot fully replicate the collective experience of a face‐to‐face group activity, RiM's move online had been necessary, well‐managed and beneficial:

I'm glad I did go online because although it's not perhaps as good, there is no reason why I couldn't and that's the way the world was going, and it made sense to continue as opposed to just completely stop. (Carrie)

#### 4.3.2 The support I received from others

The skills which RiM taught and the strategies which RiM facilitated formed only part of the overall experience. Equally as important to students was the support that they received from fellow students, peer trainers and staff alike. Students found this support particularly valuable during the pandemic.

##### Being with others who'd been through similar showed me that some people do understand

Students described the great extent to which they valued the shared experiences of other peer trainers and fellow students:

It's very helpful to have Angela … her experience and her journey and her commitment to what she does was the most inspiring I have to say because it was not professional. I think she was a major pull to the course. (Julia)

When you started talking to people you realised that they were there for whatever reason, we all had similar backgrounds or mental health problems. It was great … people like me (Sam)

Students no longer felt the isolation and sense of alienation which had previously characterized their experience of living with mental health struggles. Julia described how the mutual understanding which existed between students meant that group members listened to one another more attentively, engaged with one another's recovery journeys more intently and took collective responsibility for the group process:

Being able to listen … I've never done therapy in a group like that in terms of recovery … It was a new thing for me to be part of a group rather than having individual sessions. I think that is very valuable indeed. I like that a lot, having other people involved in the process … the responsibility of being present, of helping others so it's more about what you can give, contribute, that I thought was valuable. (Julia)

##### The peer support helped us to develop and learn

This intensity and mutuality of peer support not only benefitted the collective group process but strengthened the ability of the group to learn collaboratively and to put their learning into practice together:

The discussion always opened things up and not always in the directions I was anticipating, and I think that was a huge advantage because I think each of us could contribute different thoughts and it actually extended your understanding … One of the other attendees on the course said, ‘This is a really good book, 10 things you can learn from Aristotle’. … and it does mention the way to a good life is by helping others. (Joe)

The cohesion of the group meant that students felt motivated to persevere with the recovery journey, even when life felt difficult:

I felt very, very nervous when my nurse said that I had been discharged from Early Intervention [in Psychosis] but I knew I still had RiM there and I'm nowhere near ready to give it up. They're [also] wanting to manage their own mental health and they want to try and get themselves better, maybe not better but more of a way of learning how to cope and see things in different ways and try different things. (Nancy)

##### The facilitators' teamwork modelled the value of being with others

Students also spoke positively of the staff team. Throughout the Covid‐19 pandemic, AR has intentionally shared increasingly fewer details of her personal recovery journey with RiM groups, and has provided progressively less one‐to‐one support to individual students, delegating this role to newer peer trainers to model the RC emphasis on moving forward. Students commented upon the collaboration and teamwork they observed with the RiM leadership, observing how AR's lived experience was accorded parity of authority and credibility with the information provided by the OTs:

I remember all the leaders. There wasn't just one, they all contributed their strengths and their experience … I thought these were good, caring people that I was happy to go to any future courses with them leading them. (Sam)

This teamwork modelled and reinforced for students what RiM was teaching them about the value of being with others and of being a group:

I'm the youngest in the group but actually that doesn't make any difference, and everyone is really supportive and we ask … for one another's email addresses at the end of the course so actually we have communicated and it's nice to be able to do that I think…. (Heidi)

Students attributed the success of RiM's move to online and socially distanced methods to the strength of relationships between staff and peer trainers, and to the confidence and ease with which facilitators worked together.

##### Being supported during lockdown

Particularly during Covid‐19, students valued the opportunity they provided for conversation, and the connectedness they offered to those who could not attend the socially distanced outdoor sessions:

[online] coffee mornings, they were good. Coffee Catch‐Ups. It was good to see [name] because I hadn't seen her for quite a while … I don't see why [we shouldn't keep them going after Covid] … maybe you won't need to do it so much but I think it would be nice. (Sam)

Students spoke equally positively about the socially distanced outdoor activities. These sessions provided opportunities for peer support, continuity in maintaining face‐to‐face relationships and respite from the boredom and isolation of lockdown. Students described how being together in the natural environment helped with anxiety and reinforced mindfulness skills, and how greatly they enjoyed these sessions. All spoke of a desire to make these sessions as inclusive as possible, though the ‘groups of six’ rule across England and differing lockdown levels in adjacent counties sometimes hindered accessibility.

#### 4.3.3 Becoming a part of something bigger

Students found this wider sense of connectedness and the most holistic sense of community of particular value because of how the pandemic had disrupted their lives. All of the students reported that they had benefitted from the weekly newsletters, both because they enjoyed the content and because it helped them feel a meaningful part of RiM:

On Tuesday this week I went onto the Coffee Catch‐Up and I was telling them that actually I felt that RiM and all the support I was getting even during Covid with all the newsletters and everything, that I was resilient. The newsletters have been really nice when we were in full lockdown last year … I contributed to the students' corner a few times and that was so lovely, seeing my pictures or the tricks that you taught me to do. (Sam)

This finding resonated closely with feedback which students had offered the RiM team. When introducing the newsletter, staff had expressed concern that students might find such regular correspondence from RiM intrusive; instead, students had told them that they welcomed and felt motivated by this contact. Beyond the sense of student community being developed by online and socially distanced outdoor activities, students described how RiM had helped them to feel more connected to the natural environment and with the people and activities present in their local area:

One of the things I've done in lockdown is look after a dog through ‘Borrow my Doggy’ and have him five days a week basically. I always think when I go out for a walk with him, after I've done [an online] coffee morning, whatever we've discussed in that session I find I think about and gives me good thinking time … more of a mindfulness time. (Carrie)

Boxes 2 and 3 present the case studies of Heidi and Joe, two of the students whose interviews informed the qualitative analysis of these findings.

Box 2 **A student's journey: Heidi**

Heidi works with racehorses, but is currently off work following a series of work‐related injuries. One of her physiotherapists once had a similar injury to hers, and it was hearing about his experience that provided her with an introduction to the benefits of peer support. Heidi has struggled with her mental health for some time, and has had several therapists, one of whom, due to Covid, she has seen entirely on Zoom. Whereas she has always found therapy helpful, she has found that RiM has taught her a lot of things that therapy has not, particular in terms of the practical skills for well‐being.

So far, Heidi has been the youngest student in both of the online courses she has attended. This does not matter to her at all—instead, she has learned a lot from their wisdom and life experience—but she does worry that some of the older students who will not have grown up with computers may have found the move to online classes difficult. She has particularly benefitted from how confidently AR and the peer trainers always speak about the problems they have had—not just for the learning and inspiration of listening to them, but because their openness makes her feel that she herself can say anything without being judged. This feeling of acceptance is important to her, because she worries that her head injury might have affected her ability to remember what she has said already.

Heidi has found lockdown extremely difficult, especially because it coincided with her accident. She has always been a very sociable person, and her injury had left her unable to drive to meet people outdoors. Before lockdown, she had relied upon the sunshine abroad to help her mental state through each winter, and she struggled with not being able to travel last winter. However, RiM has really helped her to cope, because the morning sessions have helped her to get going for the day. She is writing in her journal a lot at the moment, which is something else she learned from the classes.

It was Heidi's birthday recently, and she felt hugely touched that the class remembered and wanted to celebrate it with her during the session. Now that the lockdown is easing, she is beginning to meet other students for walks and coffees, which is something she greatly enjoys. Heidi suggests that, as RiM returns to face‐to‐face classes, the group numbers are kept small initially, so that students do not find the transition too overwhelming.

Box 3 **A student's journey: Joe**

Joe is a recently retired geologist who is fond of the natural world and deeply committed to his wife and children. Joe first realised that he might benefit from help with his thoughts and moods during the first lockdown when one of his daughters told him that she sometimes felt that he was not really with her when she was talking to him. Joe reflected privately upon this and became concerned that, although he had always provided his children with a great deal of practical support, he may not have offered them as much emotional support. This same daughter then heard about RiM, and suggested to Joe that he sign up for a course.

Joe contacted the administrator, whom he found hugely helpful in explaining how RiM works. He joined a socially distanced Welcome to Recovery course in Newbury in September 2020, and then an online Five Ways to Wellbeing in November 2020. He liked being in the group, and appreciated the honest wisdom of Angela, the professionalism of staff and the advice and book recommendations of peer trainers and other students. Joe found the courses beneficial because they taught him to understand and to take control of his difficult thoughts, and that this improved his mood. He has found mindfulness an especially useful skill to have learned.

Joe has found the Covid pandemic stressful because of the life plans that it had disrupted, and because of the differences of opinion within his family and social circle that it had exposed. However, he believes that the increased use of online technologies has been a positive thing, and has many ideas for how RiM could deliver more virtual courses. He is concerned about how, even in this day and age, some working‐age people still worry that getting involved with organizations such as RiM will harm their career prospects. He would love to encourage local employers to partner with RiM to encourage more people to seek help early.

## 5 DISCUSSION

### 5.1 Synthesis and summary of quantitative and qualitative findings

This research found that RiM courses have brought significant positive changes in students' mental well‐being, at least to those who completed the W2R course. The Covid‐19 pandemic has affected the entire lives of everyone at RiM, and many students and peer trainers have found the past 2 years immensely difficult. Nevertheless, students have welcomed the way that RiM has adapted to offering online and socially distanced outdoor activities. In particular, students have valued the way that RiM has helped them to feel connected to others and to their local community. As found by Rapisarda et al.'s^29^ evaluation of outcomes produced by online RCs during Covid‐19, this study found that RiM students derived the most significant benefit during the earliest stages of their RC journey. Similar to Rapisarda et al.'s^29^ findings that these benefits were maintained to a statistically significant threshold with regard to anxiety, the current study identified a sustained improvement to students' overall mental well‐being up to a 3‐month follow‐up.

Students felt empowered to set their own personal goals. These reflect individuals' aspirations in developing themselves and their interpersonal relationships, and in resolving mental health difficulties which had been preventing them from living their lives to the full. Considering the comparatively poor mental wellbeing and probably high needs these students had at baseline, their own subjective perception of achieving their personal goal over the RiM course is particularly positive. Additionally, many students felt sufficiently confident to set goals that they could not necessarily easily attain. Particularly for those older students who had been treated for many years in traditional mental health settings which focused on ‘maintenance’ rather than ‘recovery’, this level of ambition reflects a paradigm shift and an embodiment of RiM's recovery ethos.

### 5.2 The originality and value of study findings

#### 5.2.1 What this research adds

This research found RiM to benefit the mental health of its students in two ways: firstly, through its curriculum of toolkit skills; and secondly, through the sense of belonging that its ‘hidden curriculum’ of social learning and empowerment promotes. Whereas the educational concept of the hidden curriculum has already been identified as of value to the RC movement,^12^ this study is the first to explore specifically what an RC hidden curriculum consists of and teaches, and the first to interrogate in depth how and why an RC hidden curriculum benefits and improves student mental health.

Within the RC model, peer trainers have long been regarded as key facilitators of co‐production.^11^, ^13^ Whereas this study very much endorsed the co‐productive value of RiM's peer trainers, this research found that student co‐production is similarly important, both in improving individual mental health outcomes and in maximizing the effectiveness and accessibility of RiM itself, both before and during the Covid‐19 pandemic.

#### 5.2.2 The hidden curriculum

As educational establishments rather than mental health providers, RCs aim to teach and aim to empower students through what they teach.^11^ This research found that students benefitted from RiM both as a result of the ‘toolkit’ skills they learned from courses, and because of their less tangible experience of simply being part of RiM. Most previous studies of RC outcomes have focused on these more measurable and more readily articulated skills.^18^, ^20^, ^22^, ^38^ King et al.^39^ found that students often find skills‐based content more accessible when delivered in an RC than within a more clinical or hierarchical mental health setting. During the Covid‐19 pandemic, Rapisarda et al.^29^ found that students experienced skills‐based courses as more engaging than other online courses, with lower levels of student attrition also facilitating demonstrable quantitative validity.

Oh^40^ has criticized this disproportionate emphasis on evaluating RC skills teaching, highlighting how ‘banking’ knowledge is anathema to the pedagogical theory underpinning RCs.^11^ This study explores not only how RiM teaches skills and strategies which students can use to improve their own wellbeing, but also how it promotes the concept and possibility of recovery and facilitates an atmosphere and a culture within which students can empower and learn from one another. Being among people on similar journeys, they found, was equally as empowering as the content of what the RiM courses directly taught. The ‘hidden curriculum’^12^ of belongingness and co‐production proved as effective as the curriculum of the recovery skills taught.

Repper and Perkins have previously cited Freire's model of the value of the hidden curriculum^41^ to RCs.^12^ However, Roper et al.^42^ caution that hidden curriculums, if not underpinning meaningfully emancipatory, social and active models of learning may re‐enforce hierarchies and power differentials which hinder togetherness and co‐production. Neve and Collet^43^ highlight how, in the absence of a clear educational philosophy, hidden curriculums may deliver confounding messages which hinder empowerment.

This research found RiM's hidden curriculum as of benefit to students because it encouraged them to learn from one another as well as from staff comprising peer‐trainers leading RiM and its courses, and provided opportunities to use their newly acquired learning as a means to empower themselves. In particular, students described the sense of belongingness promoted by RiM's hidden curriculum as of immense benefit to their mental health. Being together with others undertaking similar journeys through mental ill‐health, isolation and uncertainty, and realizing collectively that the concept and possibility of recovery could offer self‐determination and hope, helped students to feel more comfortable with being themselves and being part of their communities.

#### 5.2.3 Co‐production

Central to the RC model is the role of the peer trainers, former students who make use of their lived experience and recovery to co‐deliver courses and to provide support to students.^11^ In this research, RiM students spoke highly of how AR and the peer trainers drew upon their knowledge and experience. Students spoke particularly highly of AR's delegation and moving forward, which meant that the peer trainers and staff encouraged an atmosphere in which students could learn from, support and empower one another.

Notwithstanding the RC model's ethos of co‐production and of valuing lived experience as equal to professional experience, most RCs are led and managed by mental health professionals, and many RCs often employ their peer trainers on lower pay grades and less secure contracts than the professional colleagues with whom they share equal responsibility.^14^ RiM has succeeded to some extent in addressing the power differentials between professionals and peer trainers by continuing to be led by AR identifying as a person with lived experience, and by remunerating non‐NHS OTs and peer trainers in the same way as one another. However, the team remains mindful that, whereas zero‐hour contracts may offer peer trainers the flexibility they need to remain employed, zero‐hour contracts may also reinforce the economic marginalisation of people with mental ill‐health.^44^ RiM is aware progress is still needed.

This study identified co‐production occurring amongst students and peer trainers whereas other RC evaluations have identified co‐production as the preserve of peer trainers,^15^, ^45^ particularly during periods of service development and change. Bester et al.^24^ and Ali et al.^46^ identify this as primarily due to the challenges of embedding RC activity within a host organization more accustomed to engaging co‐productively with specific ‘experts by experience’ rather than with a cohort of service users. As an independent RC, RiM has never faced this challenge. This research found, perhaps as a consequence of this independence, that RiM students as well as peer trainers are engaged in meaningful co‐production. By promoting co‐production amongst students, RiM, therefore, demonstrates the benefits of close fidelity to the RC ethos of empowerment not only for the sake of involvement but as a vehicle to leadership and change.^11^, ^13^ Crowther et al.^17^ and McGregor et al.^12^ both assert that student co‐production can be most meaningfully sustained in independent RCs.

### 5.3 Strengths and limitations

Even before Covid‐19, research into RCs has faced a number of unique challenges in demonstrating their validity and reliability.^18^, ^26^ Because RCs, unlike most mental health services, operate on a self‐referral and opt‐in basis,^11^ people who participate in research into RCs tend to be those who have benefitted from them, meaning that research into RCs tends to generate positive findings. This study sought to mitigate against this bias by purposively seeking to recruit students who RiM were aware were not finding their participation unproblematic. Nevertheless, because RCs have a strong ethos of encouraging students and peer trainers to move forward with their lives,^11^ they do not necessarily retain contact details for successful and satisfied former students who might generate the most positive findings.

Given the relative homogeneity of students in terms of gender, age range and ethnicity — mostly White women in their 40s and 50s — study results may have limited generalisability for other populations, such as men and people from ethnic minority backgrounds. Like all research undertaken during 2020 and 2021, the Covid‐19 pandemic posed unforeseen obstacles. Fewer potential students might have joined RiM during such time, compared to a similar duration pre‐dating the pandemic. Further, fewer students participated in the follow‐up data collection, and a higher frequency of non‐attendance during this time caused a loss of follow‐up data. Researchers believe some non‐attendance or attrition was partly due to interruptions in students' engagement with RiM as much as other aspects of day‐to‐day life throughout the pandemic. Some may be due to data collection via post or email (with phone support) instead of in‐person administration.

In view of the limited unmatched sample data across time points, the planned statistical analyses became inappropriate. Instead, a descriptive approach was used to chart the changes in students' well‐being outcomes (using WEMWBS) over time, and results should be interpreted with caution. Pandemic restrictions meant that interviews had to be rescheduled and conducted online, potentially jeopardizing the alliance between interviewers and interviewees. Given the pandemic is known to have been particularly detrimental to individuals with pre‐existing mental ill‐health concerns,^5^, ^6^ findings therefore should not be taken as overall RiM outcomes.

## 6 CONCLUSION

Despite the challenges posed by Covid‐19, RiM continued throughout the pandemic to uphold its ‘hidden curriculum’ of belongingness and co‐production, and its emphasis on co‐learning and co‐production. Future research might consider what other RCs have learned from the Covid‐19 pandemic, and how this might inform co‐production to improve recovery outcomes. This research emphasises co‐production as not only a tool for collective empowerment or service improvement but as a valuable skill for personal mental health recovery. In so doing, this research demonstrates RiM continued fidelity to the RC model of co‐production as crucial and integral to its success. Even when operating until the most unforeseen or challenging of conditions,^8^ RCs should always endeavour to prioritize and maintain co‐production.

## AUTHOR CONTRIBUTIONS

Angela Ryan, Cath Hensby, Fiona Habermehl, Sarah Burton and Jacqueline Sin, as a group, conceived and designed the study including the development of the participant‐facing materials, the interview topic guide and the goal‐statement measure. Heather Yoeli, Angela Ryan, Cath Hensby, Fiona Habermehl and Sarah Burton led data collection. Heather Yoeli and Jacqueline Sin led the data analysis, in consultation with Angela Ryan, Cath Hensby, Fiona Habermehl and Sarah Burton. All authors contributed to the interpretation of the analysed results. Heather Yoeli drafted the paper with support from Jacqueline Sin. All authors commented and contributed to the revisions. All authors read and approved the final version and its submission.

## CONFLICT OF INTEREST

The authors declare no conflict of interest.

## Data Availability

The anonymized data that support the findings of this study are available on request from the corresponding author. The data are not publicly available due to privacy and ethical restrictions.
